# Structural and theoretical studies of 4-chloro-2-methyl-6-oxo-3,6-dideuteropyrimidin-1-ium chloride (*d* ^6^)

**DOI:** 10.1107/S205698902100270X

**Published:** 2021-03-19

**Authors:** Ray J. Butcher, Andrew P. Purdy, Sean A. Fischer, Daniel Gunlycke

**Affiliations:** a Department of Chemistry, Howard University, 525 College Street NW, Washington DC 20059, USA; b Chemistry Division, Code 6100, Naval Research Laboratory, 4555 Overlook Av, SW, Washington DC 20375-5342, USA; c Chemistry Division, Code 6189, Naval Research Laboratory, 4555 Overlook Av, SW, Washington DC 20375-5342, USA

**Keywords:** crystal structure, pyrimidinium cation, distorted *sp*^2^ C

## Abstract

The title compound, C_5_D_6_ClN_2_O^+^·Cl^−^, crystallizes in the ortho­rhom­bic space group, *Pbcm*, and consists of a 4-chloro-2-methyl-6-oxo-3,6-di­hydro­pyrimidin-1-ium cation and a chloride anion where both moieties lie on a crystallographic mirror. The cation is disordered and was refined as two equivalent forms with occupancies of 0.750 (4)/0.250 (4), while the chloride anion is triply disordered with occupancies of 0.774 (12), 0.12 (2), and 0.11 (2). Unusually, the bond angles around the C=O unit range from 127.2 (6) to 115.2 (3)° and similar angles have been found in other structures containing a 6-oxo-3,6-di­hydro­pyrimidin-1-ium cation, including the monclinic polymorph of the title compound, which crystallizes in the monoclinic space group *P*2_1_/*c* [Kawai *et al.* (1973 ▸). *Cryst. Struct. Comm.* **2**, 663–666]. The cations and anions pack into sheets in the *ab* plane linked by N—H⋯Cl hydrogen bonds as well as C—H⋯O and Cl⋯O inter­actions. In graph-set notation, these form *R* ^3^_3_(11) and *R* ^3^_2_(9) rings. Theoretical calculations seem to indicate that the reason for the unusual angles at the *sp* ^2^ C is the electrostatic inter­action between the oxygen atom and the adjacent N—H hydrogen.

## Abstract 2

The crystal structure of 4-chloro-2-methyl-6-oxo-3,6-dideuteropyrimidin-1-ium chloride exhibits unusual angles about an *sp* ^2^ C atom, which are confirmed by theoretical calculations.

## Chemical context   

Heterocycles containing the pyrimidine moiety are of great inter­est because they constitute an important class of natural and non-natural products, many of which exhibit useful biological activities and clinical applications (Brown, 1984 ▸; Elderfield, 1957 ▸). Substituted purines and pyrimidines occur very widely in living organisms and were some of the first compounds studied by organic chemists (Bruice, 2007 ▸).

The presence of the pyrimidine base in thymine, cytosine, and uracil, which are the essential building blocks of nucleic acids DNA and RNA, is one possible reason for their widespread therapeutic applications. Pyrimidines represent one of the most active classes of compounds, possessing a wide spectrum of biological activities such as significant *in vitro* activity against unrelated DNA and RNA viruses including polio herpes viruses, and diuretic, anti­tumor, anti-HIV, and cardiovascular (Kappe, 1993 ▸) activity. In addition to this, various analogs of pyrimidines have been found to possess anti­bacterial (Sharma *et al.*, 2004 ▸; Prakash *et al.*, 2004 ▸; Botta *et al.*, 1992 ▸; Cieplik *et al.*, 2015 ▸), anti­fungal (Agarwal *et al.*, 2000 ▸; Oliver *et al.*, 2016 ▸), anti­leishmanial (Ram *et al.*, 1992 ▸; Alptuzun *et al.*, 2013 ▸), anti-inflammatory (Amir *et al.*, 2007 ▸; Sondhi *et al.*, 2008 ▸), analgesic (Vega *et al.*, 1990 ▸; Gupta *et al.*, 2011 ▸), anti­hypertensive (Hannah & Stevens, 2003 ▸; Rana *et al.*, 2004 ▸; Alam *et al.*, 2010 ▸), anti­pyretic (Smith & Kan, 1964 ▸; El-Sharkawy *et al.*, 2018 ▸), anti­viral (Balzarini & McGuigan, 2002 ▸; Nasr & Gineinah, 2002 ▸), anti­diabetic (Lee *et al.*, 2005 ▸; Reddy *et al.*, 2019 ▸), anti­allergic (Juby *et al.*, 1979 ▸; Gupta *et al.*, 1995 ▸), anti­convulsant (Gupta *et al.*, 1994 ▸; Shaquiquzzaman *et al.*, 2012 ▸), anti­oxidant (Krivonogov, *et al.*, 2001 ▸; Abu-Hashem *et al.*, 2010 ▸, 2011 ▸), anti­histaminic (Prasad & Rahaman, 2008 ▸; Rahaman *et al.*, 2009 ▸), herbicidal (Nezu *et al.*, 1996 ▸; Li *et al.*, 2018 ▸), and anti­cancer activities (Abu-Hashem *et al.*, 2010 ▸, 2011 ▸; Xie *et al.*, 2009 ▸; Kaldrikyan *et al.*, 2000 ▸; Mohamed *et al.*, 2013 ▸) and many pyrimidine derivatives are reported to possess potential central nervous system (CNS) depressant properties (Rodrigues *et al.*, 2005 ▸; Tani *et al.*, 1979 ▸; Kimura *et al.*, 1993 ▸) and also act as calcium channel blockers (Kumar *et al.*, 2002 ▸; Ortner & Striessnig, 2016 ▸). Thus, in view of this extensive biochemical activity of pyrimidines and their derivatives, much effort has been expended on the structural study of both pyrimidines and their cations.
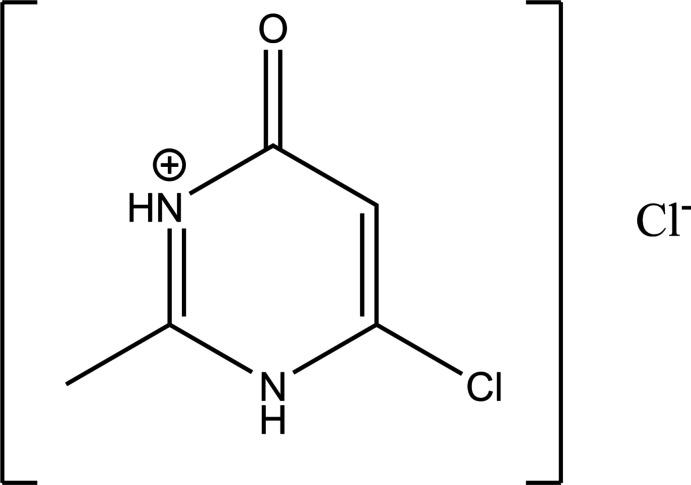


## Structural commentary and database survey   

**Figure 1:**
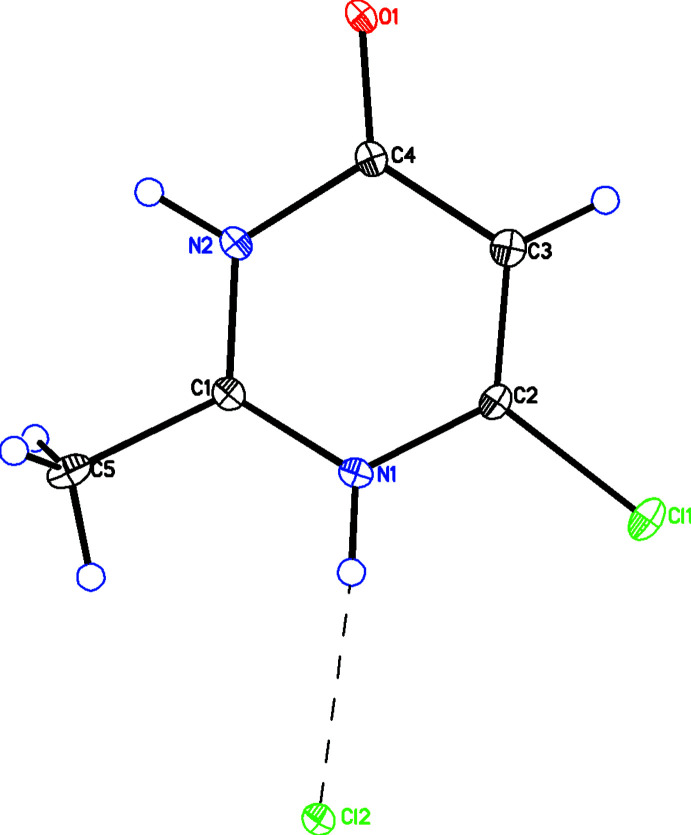
Diagram showing the cation and anion and the atom-numbering scheme (only the major component of the disorder is shown) with atomic displacement parameters drawn at the 30% probability level. The N—H⋯Cl hydrogen bond is shown by a dashed line.

The title compound, [C_5_D_6_ClN_2_O]^+^Cl^−^, **1**, crystallizes in the ortho­rhom­bic space group, *Pbcm*, unlike its polymorph, **2** (Kawai *et al.*, 1973 ▸), which crystallizes in the monoclinic space group *P*2_1_/*c*. It consists of a 4-chloro-2-methyl-6-oxo-3,6-di­hydro­pyrimidin-1-ium cation and a chloride anion (Fig. 1 ▸). Since both moieties lie on a crystallographic mirror plane, the cation is strictly planar. The cation is disordered over two equivalent conformations (both of which lie on the mirror plane) with occupancies of 0.750 (4)/0.250 (4) while the chloride anion is triply disordered with occupancies of 0.774 (12), 0.12 (2), and 0.11 (2). The C—C, C—N, and C=O metrical parameters of the 6-oxo-3,6-di­hydro­pyrimidin-1-ium skeleton for the two polymorphs are similar and both exhibit unusual bond angles for the ketonic moiety. The values for C3—C4—O1, N2—C4—O1, and N2—C4—C3 are 127.2 (6), 117.6 (6), and 115.2 (3)° for **1** and 126.1 (9), 118.2 (8), and 115.7 (8)° for **2**.

In view of the unusual values for these bond angles, a search was made of the Cambridge Structural Database [CSD version 5.41 (November 2019); Groom *et al.*, 2016 ▸] for structures containing a 6-oxo-3,6-di­hydro­pyrimidin-1-ium skeleton, which yielded 52 independent observations. A statistical analysis of the values for corresponding angles gave values of 126.7 (7), 118.8 (8), and 115.3 (10)°. An analysis of both lengths also revealed the similarity in all these derivatives. In all cases, the longest bond was C3—C4 which is 1.430 (7) Å in **1** and 1.430 (12) Å on average, while the second longest bond was N2—C4 at 1.402 (6) Å for **1** and 1.397 (10) Å on average. In fact, all the metrical parameters for the 6-oxo-3,6-di­hydro­pyrimidin-1-ium skeleton are in agreement with average values. One reason to be considered for the unusual values for the C3—C4—O1, N2—C4—O1, and N2—C4—C3 angles is this difference in C3—C4 and C4—N2 distances, which would tilt the carbonyl moiety towards N2. However, there are examples where the lengths of these two distances are reversed [ACEYUD (Mu­thiah *et al.*, 2004 ▸), EHAPOV (Tapmeyer & Prill, 2019 ▸), SUZFOJ (Suleiman Gwaram *et al.*, 2010 ▸)], but the same trend in angles prevails.

In light of these unusual bond angles for an *sp* ^2^ C atom, a theoretical analysis of the cation was undertaken. The geometries of the isolated cation, two neutral variants, and a tautomer of the cation were optimized using the PBE0 exchange-correlation functional (Adamo & Barone, 1999 ▸; Perdew *et al.*, 1996 ▸) and aug-cc-pVTZ basis set (Dunning, 1989 ▸; Kendall *et al.*, 1992 ▸; Woon & Dunning 1993 ▸; Davidson, 1996 ▸) *via NWChem* (Aprà *et al.*, 2020 ▸). The geometry of the cation was also optimized as a scan was made of the nuclear charge of the hydrogen bound to N2.

**Figure 2:**
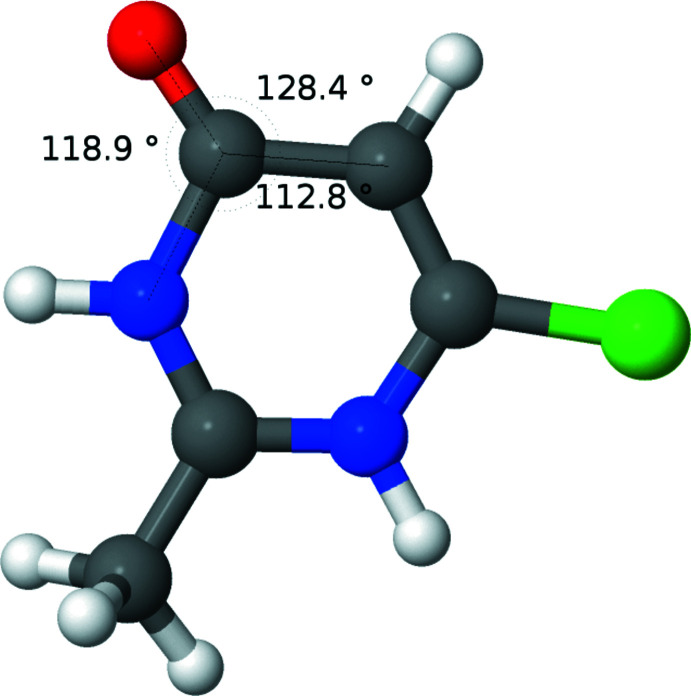
Diagram showing the results of calculations for the cation, **1**. Relevant angles are displayed.

**Figure 3:**
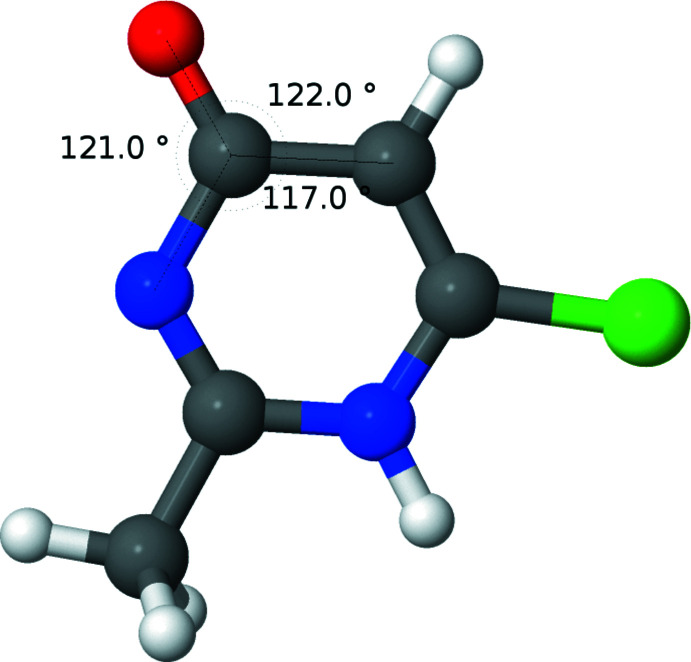
Diagram showing the results of calculations for the neutral mol­ecule, **3** (tautomer 1). Relevant angles are displayed.

**Figure 4:**
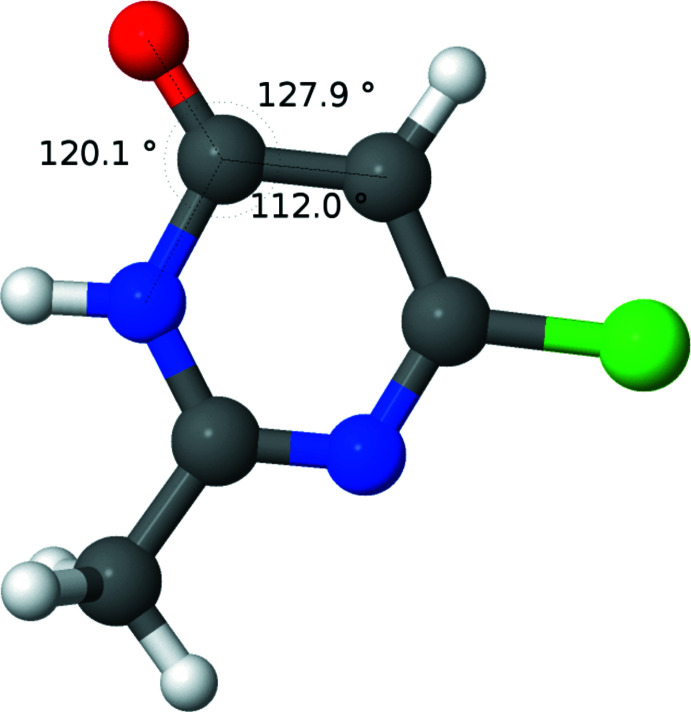
Diagram showing the results of calculations for the neutral mol­ecule, **4** (tautomer 2, with the N—H group adjacent to the C=O bond). Relevant angles are displayed.

**Figure 5:**
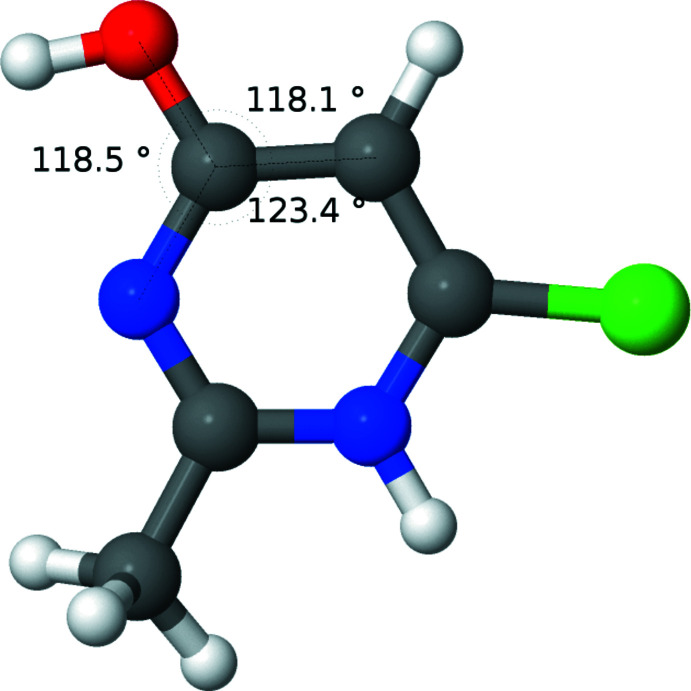
Diagram showing the results of calculations for **5**, a tautomer of the cation. Relevant angles are displayed.

**Figure 6:**
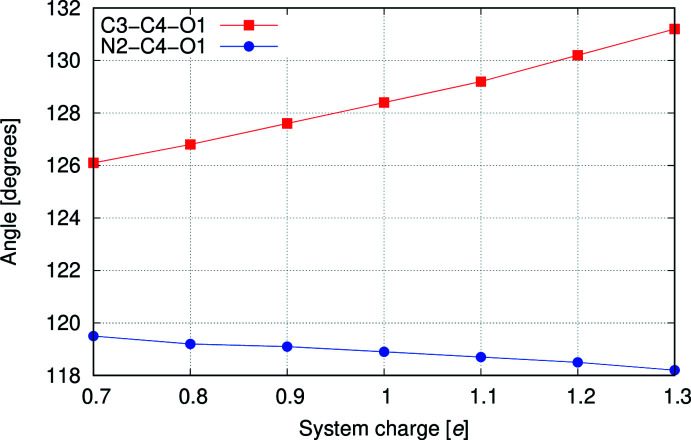
Diagram showing a plot of the variation in the C—C—O and N—C—O angles around the *sp* ^2^ C atom as the nuclear charge of the hydrogen attached to the nitro­gen is varied while keeping all other nuclear charges fixed at their normal values and keeping the number of electrons fixed.

Figs. 2 ▸–5 ▸ ▸ ▸ show the optimized geometries for the cation **1**, two neutral structures, **3** and **4**, which are tautomers of each other, and a tautomer of the cation, **5**. When N2 is protonated, as in **1** (Fig. 2 ▸) and **4** (Fig. 4 ▸), the carbonyl moiety is tilted towards N2. When N2 is not protonated, as in **3** (Fig. 3 ▸) and **5** (Fig. 5 ▸), the carbonyl moiety assumes a normal orientation for an *sp* ^2^ C atom. This suggests an electrostatic inter­action between oxygen and hydrogen may be responsible for the unusual angles. To explore this further, the geometry of **1** was optimized as the nuclear charge of the hydrogen bound to N2 was scanned from 0.7 to 1.3 *e*. As can be seen from the plot (Fig. 6 ▸), the two angles converge with decreasing nuclear charge on the hydrogen and diverge with increasing nuclear charge. This lends further support to the idea that the origin of the angle difference is an electrostatic inter­action between the O1 and the hydrogen on N2.

## Supra­molecular features   

**Figure 7:**
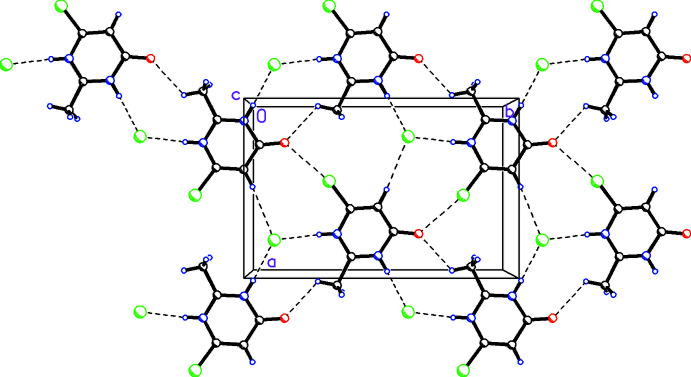
The packing viewed along the *c* axis showing how the cations and anions pack into sheets in the *ab* plane linked by N—H⋯Cl hydrogen bonds and Cl⋯O and weak C—H⋯O inter­actions, forming 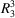
(11) and 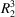
(9) rings.

In the crystal, the cations and anions pack into sheets in the *ab* plane linked by N—H⋯Cl hydrogen bonds, as well as Cl⋯O and weak C—H⋯O inter­actions (Table 1 ▸). In graph-set notation (Etter *et al.*, 1990 ▸), these make 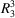
(11) and 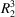
(9) rings as seen in Fig. 7 ▸. Inter­estingly there are no N—H⋯O hydrogen bonds.

## Synthesis and crystallization   

Inside a dry box, one side of an H-tube (with no filter between the sides) was charged with 250 mg triphosgene (Aldrich) and the other side was loaded with 20 mg tetra­methyl­ammonium chloride in 3 mL dry tetra­glyme. Once attached to a vacuum line with Cajon flexible tubing, the components were mixed and the phosgene was collected in a vacuum trap. In one NMR tube, 0.36 mmol of phosgene were measured on the vacuum line, condensed into 0.75 mL of dry CD_3_CN, and the tube was sealed as an NMR reference. In another tube, 0.36 mmol of phosgene was condensed onto 0.06 g (0.20 mmol) of silver oxalate in CD_3_CN and the tube was sealed to attempt to prepare a CO_2_ polymer. Upon warming, the ^13^C NMR of the reaction tube showed gaseous CO_2_ and solvent only. After standing unobserved for three years, the reference tube was observed to be filled with crystals of the title compound, which is completely insoluble in aceto­nitrile, and the tube was opened in a drybox to keep the crystals dry. The ^13^C{^1^H} NMR spectrum of the crystals in D_2_O (DSS ref) is 167.07 (*s*), 164.11 (*s*), 161.28 (*s*), 113.05 (C3, *t*, ^1^ *J*_C–D_ = 27.5 Hz) , 22.35 (CD_3_, septet, ^1^ *J*_C–D_ = 19.8 Hz) . The previous report (Yanagida *et al.*, 1968 ▸) involved a reaction of phosgene, CH_3_CN and HCl at 338 K. In contrast to a previous report for the structure of the monoclinic polymorph (Kawai *et al.*, 1973 ▸), all crystals had the same habit and appearance and one suitable for X-ray diffraction studies was chosen for further study.

## Refinement   

Crystal data, data collection and structure refinement details are summarized in Table 2 ▸. The cation is disordered and was refined as two equivalent forms with occupancies of 0.750 (4)/0.250 (4), while the chloride anion is triply disordered with occupancies of 0.774 (12), 0.12 (2), and 0.11 (2). The locations of all deuterium atoms for the major component except one attached to N1 were located in difference-Fourier maps and refined in idealized positions using a riding model with atomic displacement parameters of *U*_iso_(D) = 1.2*U*_eq_(C, N) [1.5*U*_eq_(C) for CD_3_], and C—D and N—D distances of 0.95 and 0.88 Å, respectively. The deuterium atoms for the methyl substituent were refined isotropically.

## Supplementary Material

Crystal structure: contains datablock(s) I. DOI: 10.1107/S205698902100270X/ru2073sup1.cif

Structure factors: contains datablock(s) I. DOI: 10.1107/S205698902100270X/ru2073Isup2.hkl

Supporting information file. DOI: 10.1107/S205698902100270X/ru2073Isup3.cml

CCDC reference: 2069792

Additional supporting information:  crystallographic information; 3D view; checkCIF report

## Figures and Tables

**Table 1:** Hydrogen-bond geometry (Å, °)

*D*—H⋯*A*	*D*—H	H⋯*A*	*D*⋯*A*	*D*—H⋯*A*
N1—D1*A*⋯Cl2^i^	0.88	2.23	3.103 (4)	175
N2—D2*A*⋯Cl2^ii^	0.88	2.24	3.119 (6)	178
C3—D3*A*⋯Cl2^iii^	0.95	2.82	3.769 (7)	175
C5—D5*B*⋯Cl2^i^	0.95 (1)	2.96 (1)	3.798 (6)	148 (1)
C5—D5*B*⋯O1^iv^	0.95 (1)	2.44 (1)	3.040 (8)	121 (1)
N1*A*—D1*AA*⋯Cl2*A* ^i^	0.88	2.37	3.24 (3)	172
N2*A*—D2*AA*⋯Cl2*A* ^iii^	0.88	2.03	2.91 (4)	177
C3*A*—D3*AA*⋯Cl2*A* ^ii^	0.95	2.94	3.88 (3)	171
C5*A*—D5*C*⋯O1*A* ^v^	0.95 (1)	2.36 (2)	2.985 (16)	123 (1)
C5*A*—D5*D*⋯O1*A* ^vi^	0.95 (1)	2.66 (1)	3.582 (9)	163 (1)

Symmetry codes: (i) x, -y+{\script{1\over 2}}, -z+1; (ii) -x+2, -y, -z+1; (iii) -x+1, -y, -z+1; (iv) -x+2, y+{\script{1\over 2}}, z; (v) -x+1, y+{\script{1\over 2}}, z; (vi) -x+1, -y, -z.

**Table 2:** Experimental details

Crystal data	
Chemical formula	C_5_D_6_ClN_2_O^+^·Cl^−^
*M*_r_	187.05
Crystal system, space group	Orthorhombic, *P* *b* *c* *m*
Temperature (K)	100
*a*, *b*, *c* (Å)	8.6030 (4), 13.1389 (6), 6.4812 (3)
*V* (Å^3^)	732.60 (6)
*Z*	4
Radiation type	Mo *K*α
μ (mm^−1^)	0.81
Crystal size (mm)	0.25 × 0.18 × 0.06

Data collection	
Diffractometer	Bruker APEXII CCD
Absorption correction	Multi-scan (*SADABS*; Sheldrick, 1996 ▸)
*T*_min_, *T*_max_	0.635, 0.747
No. of measured, independent and observed [*I* > 2σ(*I*)] reflections	12377, 1802, 1558
*R*_int_	0.041
(sin θ/λ)_max_ (Å^−1^)	0.820

Refinement	
*R*[*F* ^2^ > 2σ(*F* ^2^)], *wR*(*F* ^2^), *S*	0.064, 0.152, 1.19
No. of reflections	1802
No. of parameters	137
No. of restraints	362
Δρ_max_, Δρ_min_ (e Å^−3^)	0.68, −1.09

Computer programs: *APEX2* (Bruker, 2005 ▸), *SAINT* (Bruker 2002 ▸), *SHELXT* (Sheldrick 2015*a* ▸), *SHELXL2018/3* (Sheldrick, 2015*b* ▸), and *SHELXTL* (Sheldrick 2008 ▸).

## supplementary crystallographic information

### Crystal data

**Table:**

C_5_D_6_ClN_2_O^+^·Cl^−^	*D*_x_ = 1.696 Mg m^−^^3^
*M**_r_* = 187.05	Mo *K*α radiation, λ = 0.71073 Å
Orthorhombic, *P**b**c**m*	Cell parameters from 8906 reflections
*a* = 8.6030 (4) Å	θ = 2.8–35.6°
*b* = 13.1389 (6) Å	µ = 0.81 mm^−^^1^
*c* = 6.4812 (3) Å	*T* = 100 K
*V* = 732.60 (6) Å^3^	Plate, pale yellow
*Z* = 4	0.25 × 0.18 × 0.06 mm
*F*(000) = 368	

### Data collection

**Table:**

Bruker APEXII CCD diffractometer	1558 reflections with *I* > 2σ(*I*)
φ and ω scans	*R*_int_ = 0.041
Absorption correction: multi-scan (SADABS; Sheldrick, 1996)	θ_max_ = 35.6°, θ_min_ = 2.8°
*T*_min_ = 0.635, *T*_max_ = 0.747	*h* = −13→14
12377 measured reflections	*k* = −21→14
1802 independent reflections	*l* = −9→10

### Refinement

**Table:**

Refinement on *F*^2^	362 restraints
Least-squares matrix: full	Primary atom site location: structure-invariant direct methods
*R*[*F*^2^ > 2σ(*F*^2^)] = 0.064	Secondary atom site location: difference Fourier map
*wR*(*F*^2^) = 0.152	*w* = 1/[σ^2^(*F*_o_^2^) + (0.0253*P*)^2^ + 2.2439*P*] where *P* = (*F*_o_^2^ + 2*F*_c_^2^)/3
*S* = 1.19	(Δ/σ)_max_ = 0.001
1802 reflections	Δρ_max_ = 0.68 e Å^−^^3^
137 parameters	Δρ_min_ = −1.09 e Å^−^^3^

### Special details

**Table:**

Geometry. All esds (except the esd in the dihedral angle between two l.s. planes) are estimated using the full covariance matrix. The cell esds are taken into account individually in the estimation of esds in distances, angles and torsion angles; correlations between esds in cell parameters are only used when they are defined by crystal symmetry. An approximate (isotropic) treatment of cell esds is used for estimating esds involving l.s. planes.

### Fractional atomic coordinates and isotropic or equivalent isotropic displacement parameters (Å^2^)

**Table:**

	*x*	*y*	*z*	*U*_iso_*/*U*_eq_	Occ. (<1)
Cl2	0.79527 (11)	0.10338 (6)	0.750000	0.0147 (2)	0.773 (4)
Cl2A	0.689 (3)	0.0986 (17)	0.750000	0.0189 (12)	0.09 (2)
Cl2B	0.714 (3)	0.1092 (13)	0.750000	0.0191 (11)	0.13 (2)
Cl1	0.46030 (12)	0.19112 (9)	0.250000	0.0235 (3)	0.747 (4)
O1	0.7595 (7)	−0.1423 (3)	0.250000	0.0247 (9)	0.747 (4)
N1	0.7600 (5)	0.1616 (3)	0.250000	0.0141 (6)	0.747 (4)
D1A	0.763535	0.228547	0.250000	0.017*	0.747 (4)
N2	0.8852 (7)	0.0087 (4)	0.250000	0.0152 (5)	0.747 (4)
D2A	0.973805	−0.024740	0.250000	0.018*	0.747 (4)
C1	0.8910 (6)	0.1091 (4)	0.250000	0.0143 (6)	0.747 (4)
C2	0.6181 (6)	0.1132 (4)	0.250000	0.0153 (6)	0.747 (4)
C3	0.6072 (8)	0.0099 (5)	0.250000	0.0185 (7)	0.747 (4)
D3A	0.508666	−0.022705	0.250000	0.022*	0.747 (4)
C4	0.7475 (9)	−0.0484 (3)	0.250000	0.0170 (6)	0.747 (4)
C5	1.0525 (9)	0.1617 (5)	0.250000	0.0233 (10)	0.747 (4)
D5A	1.0986 (14)	0.1441 (10)	0.1214 (19)	0.035*	0.747 (4)
D5B	1.0311 (14)	0.2329 (5)	0.250000	0.035*	0.747 (4)
Cl1A	1.0368 (7)	0.1850 (5)	0.250000	0.0364 (14)	0.253 (4)
O1A	0.734 (2)	−0.1471 (7)	0.250000	0.026 (2)	0.253 (4)
N1A	0.7367 (12)	0.1568 (8)	0.250000	0.0163 (11)	0.253 (4)
D1AA	0.733683	0.223774	0.250000	0.020*	0.253 (4)
N2A	0.6105 (17)	0.0044 (10)	0.250000	0.0179 (11)	0.253 (4)
D2AA	0.521578	−0.028785	0.250000	0.021*	0.253 (4)
C1A	0.6051 (12)	0.1048 (10)	0.250000	0.0168 (11)	0.253 (4)
C2A	0.8783 (12)	0.1079 (9)	0.250000	0.0164 (11)	0.253 (4)
C3A	0.8883 (18)	0.0046 (9)	0.250000	0.0168 (11)	0.253 (4)
D3AA	0.986561	−0.028400	0.250000	0.020*	0.253 (4)
C4A	0.748 (2)	−0.0533 (7)	0.250000	0.0177 (11)	0.253 (4)
C5A	0.4429 (14)	0.1574 (10)	0.250000	0.0233 (10)	0.253 (4)
D5C	0.464 (2)	0.2283 (10)	0.250000	0.035*	0.253 (4)
D5D	0.3968 (19)	0.1390 (15)	0.122 (2)	0.035*	0.253 (4)

### Atomic displacement parameters (Å^2^)

**Table:**

	*U*^11^	*U*^22^	*U*^33^	*U*^12^	*U*^13^	*U*^23^
Cl2	0.0144 (5)	0.0132 (3)	0.0164 (3)	0.0011 (3)	0.000	0.000
Cl2A	0.017 (3)	0.019 (2)	0.0204 (18)	−0.004 (2)	0.000	0.000
Cl2B	0.017 (2)	0.021 (2)	0.0193 (16)	−0.004 (2)	0.000	0.000
Cl1	0.0173 (4)	0.0233 (5)	0.0298 (5)	0.0084 (3)	0.000	0.000
O1	0.0121 (18)	0.0141 (12)	0.048 (2)	−0.0039 (10)	0.000	0.000
N1	0.0155 (12)	0.0117 (11)	0.0151 (11)	−0.0013 (9)	0.000	0.000
N2	0.0132 (11)	0.0139 (11)	0.0187 (12)	−0.0029 (9)	0.000	0.000
C1	0.0144 (12)	0.0139 (11)	0.0145 (12)	−0.0012 (10)	0.000	0.000
C2	0.0133 (12)	0.0160 (13)	0.0165 (12)	0.0030 (10)	0.000	0.000
C3	0.0161 (12)	0.0182 (13)	0.0212 (14)	0.0006 (11)	0.000	0.000
C4	0.0129 (12)	0.0158 (12)	0.0224 (13)	−0.0015 (10)	0.000	0.000
C5	0.0257 (17)	0.0149 (18)	0.029 (2)	0.0063 (13)	0.000	0.000
Cl1A	0.029 (2)	0.045 (3)	0.035 (2)	−0.005 (2)	0.000	0.000
O1A	0.018 (5)	0.017 (3)	0.044 (5)	0.001 (3)	0.000	0.000
N1A	0.018 (2)	0.014 (2)	0.016 (2)	0.0017 (18)	0.000	0.000
N2A	0.016 (2)	0.0172 (19)	0.021 (2)	0.0011 (18)	0.000	0.000
C1A	0.0154 (19)	0.017 (2)	0.018 (2)	0.0020 (18)	0.000	0.000
C2A	0.0168 (19)	0.0158 (19)	0.017 (2)	0.0003 (18)	0.000	0.000
C3A	0.015 (2)	0.0171 (19)	0.019 (2)	0.0007 (18)	0.000	0.000
C4A	0.014 (2)	0.0166 (19)	0.022 (2)	−0.0006 (18)	0.000	0.000
C5A	0.0257 (17)	0.0149 (18)	0.029 (2)	0.0063 (13)	0.000	0.000

### Geometric parameters (Å, º)

**Table:**

Cl1—C2	1.700 (5)	Cl1A—D5A	1.126 (8)
Cl1—D5D^i^	1.206 (11)	Cl1A—D5B	0.631 (10)
O1—C4	1.238 (4)	O1A—C4A	1.238 (5)
N1—C1	1.322 (6)	N1A—C1A	1.322 (7)
N1—C2	1.377 (6)	N1A—C2A	1.378 (7)
N1—D1A	0.8800	N1A—D1AA	0.8800
N2—C1	1.320 (6)	N2A—C1A	1.321 (8)
N2—C4	1.402 (6)	N2A—C4A	1.402 (8)
N2—D2A	0.8800	N2A—D2AA	0.8800
C1—C5	1.552 (9)	C1A—C5A	1.556 (10)
C2—C3	1.360 (7)	C2A—C3A	1.361 (8)
C3—C4	1.430 (7)	C3A—C4A	1.429 (9)
C3—D3A	0.9500	C3A—D3AA	0.9500
C5—D5A	0.952 (5)	C5A—D5C	0.950 (5)
C5—D5B	0.953 (5)	C5A—D5D	0.950 (5)
C5—D5A^i^	0.952 (5)	C5A—D5D^i^	0.950 (5)
Cl1A—C2A	1.698 (7)		
			
C2—Cl1—D5D^i^	91.1 (7)	D5A—Cl1A—D5B	120.9 (9)
C1—N1—C2	121.0 (3)	D5A—Cl1A—D5A^i^	96 (2)
C1—N1—D1A	119.5	D5B—Cl1A—D5A^i^	120.9 (9)
C2—N1—D1A	119.5	C1A—N1A—C2A	121.1 (5)
C1—N2—C4	124.5 (4)	C1A—N1A—D1AA	119.5
C1—N2—D2A	117.7	C2A—N1A—D1AA	119.5
C4—N2—D2A	117.7	C1A—N2A—C4A	124.7 (8)
N2—C1—N1	119.3 (4)	C1A—N2A—D2AA	117.6
N2—C1—C5	118.7 (5)	C4A—N2A—D2AA	117.6
N1—C1—C5	122.1 (4)	N2A—C1A—N1A	119.1 (8)
C3—C2—N1	121.4 (4)	N2A—C1A—C5A	118.4 (8)
C3—C2—Cl1	123.1 (4)	N1A—C1A—C5A	122.5 (8)
N1—C2—Cl1	115.4 (3)	C3A—C2A—N1A	121.4 (8)
C2—C3—C4	118.5 (5)	C3A—C2A—Cl1A	123.0 (8)
C2—C3—D3A	120.8	N1A—C2A—Cl1A	115.6 (7)
C4—C3—D3A	120.8	C2A—C3A—C4A	118.5 (8)
O1—C4—N2	117.6 (6)	C2A—C3A—D3AA	120.7
O1—C4—C3	127.2 (6)	C4A—C3A—D3AA	120.7
N2—C4—C3	115.2 (3)	O1A—C4A—N2A	117.2 (10)
C1—C5—D5A	105.3 (8)	O1A—C4A—C3A	127.7 (10)
C1—C5—D5B	105.3 (8)	N2A—C4A—C3A	115.2 (5)
D5A—C5—D5B	108.7 (8)	C1A—C5A—D5C	105.2 (9)
C1—C5—D5A^i^	105.3 (8)	C1A—C5A—D5D	105.2 (9)
D5A—C5—D5A^i^	122 (2)	D5C—C5A—D5D	109.3 (8)
D5B—C5—D5A^i^	108.7 (8)	C1A—C5A—D5D^i^	105.2 (9)
C2A—Cl1A—D5A	95.4 (7)	D5C—C5A—D5D^i^	109.3 (8)
C2A—Cl1A—D5B	122.1 (14)	D5D—C5A—D5D^i^	121 (3)
			
C4—N2—C1—N1	0.0	C4A—N2A—C1A—N1A	0.0
C4—N2—C1—C5	180.0	C4A—N2A—C1A—C5A	180.0
C2—N1—C1—N2	0.0	C2A—N1A—C1A—N2A	0.0
C2—N1—C1—C5	180.0	C2A—N1A—C1A—C5A	180.0
C1—N1—C2—C3	0.0	C1A—N1A—C2A—C3A	0.0
C1—N1—C2—Cl1	180.0	C1A—N1A—C2A—Cl1A	180.0
N1—C2—C3—C4	0.0	N1A—C2A—C3A—C4A	0.0
Cl1—C2—C3—C4	180.0	Cl1A—C2A—C3A—C4A	180.0
C1—N2—C4—O1	180.0	C1A—N2A—C4A—O1A	180.0
C1—N2—C4—C3	0.0	C1A—N2A—C4A—C3A	0.0
C2—C3—C4—O1	180.0	C2A—C3A—C4A—O1A	180.0
C2—C3—C4—N2	0.0	C2A—C3A—C4A—N2A	0.0

Symmetry code: (i) *x*, *y*, −*z*+1/2.

### Hydrogen-bond geometry (Å, º)

**Table:**

*D*—H···*A*	*D*—H	H···*A*	*D*···*A*	*D*—H···*A*
N1—D1*A*···Cl2^ii^	0.88	2.23	3.103 (4)	175
N2—D2*A*···Cl2^iii^	0.88	2.24	3.119 (6)	178
C3—D3*A*···Cl2^iv^	0.95	2.82	3.769 (7)	175
C5—D5*B*···Cl2^ii^	0.95 (1)	2.96 (1)	3.798 (6)	148 (1)
C5—D5*B*···O1^v^	0.95 (1)	2.44 (1)	3.040 (8)	121 (1)
Cl1*A*—D5*B*···O1*A*^v^	0.63 (1)	2.57 (2)	2.960 (16)	123 (1)
N1*A*—D1*AA*···Cl2*A*^ii^	0.88	2.37	3.24 (3)	172
N2*A*—D2*AA*···Cl2*A*^iv^	0.88	2.03	2.91 (4)	177
C3*A*—D3*AA*···Cl2*A*^iii^	0.95	2.94	3.88 (3)	171
C5*A*—D5*C*···O1*A*^vi^	0.95 (1)	2.36 (2)	2.985 (16)	123 (1)
C5*A*—D5*D*···O1*A*^vii^	0.95 (1)	2.66 (1)	3.582 (9)	163 (1)

Symmetry codes: (ii) *x*, −*y*+1/2, −*z*+1; (iii) −*x*+2, −*y*, −*z*+1; (iv) −*x*+1, −*y*, −*z*+1; (v) −*x*+2, *y*+1/2, *z*; (vi) −*x*+1, *y*+1/2, *z*; (vii) −*x*+1, −*y*, −*z*.
